# In vivo and in vitro efficacy of crocin against *Echinococcus multilocularis*

**DOI:** 10.1186/s13071-021-04866-4

**Published:** 2021-07-13

**Authors:** Chuanchuan Liu, Haining Fan, Lu Guan, Ri-li Ge, Lan Ma

**Affiliations:** 1 Qinghai University Affiliated Hospital, Xining, 810001 Qinghai People’s Republic of China; 2 Research Center for High Altitude Medicine, Qinghai University, Xining, 810001 Qinghai People’s Republic of China; 3 Qinghai Key Laboratory for Echinococcosis, Xining, 810001 Qinghai People’s Republic of China; 4 Qinghai Key Laboratory of Science and Technology for High Altitude Medicine, Xining, 810001 Qinghai People’s Republic of China

**Keywords:** *Echinococcus multilocularis*, Protoscoleces, Crocin, Metacestodes, Matrix metalloproteinase 2, Matrix metalloproteinase 9, Toxicity

## Abstract

**Background:**

Alveolar echinococcosis (AE) is a fatal zoonosis caused by the larvae of *Echinococcus multilocularis*. However, current chemotherapy treatment options are based on benzimidazoles [albendazole (ABZ) and mebendazole], which have limited efficacy. Therefore, novel drugs are necessary for the treatment of this disease.

**Methods:**

The anthelmintic effects of crocin were tested on *E. multilocularis* metacestodes, germinal cells and protoscoleces in vitro. Human foreskin fibroblasts (HFFs) and Reuber rat hepatoma (RH) cells were used to assess cytotoxicity. The in vivo efficacy of crocin was investigated in mice following secondary infection with *E. multilocularis*. Furthermore, collagen deposition and degradation in host tissues around the metacestodes were evaluated.

**Results:**

In vitro, crocin had a median effective concentration of 11.36 μM against cultured *E. multilocularis* metacestodes, while it reduced germinal cell viability at a median inhibitory concentration of 10.05 μM. Crocin was less toxic to HFFs and RH mammalian cell lines than to metacestodes. Transmission electron microscopy revealed that crocin treatment resulted in structural damage in the germinal layer. In addition, 60.33 ± 3.06% of protoscoleces were killed by treatment with 10 μM crocin for 7 days, indicating that crocin has a parasiticidal effect. In vivo, the metacestode weight was significantly reduced after the administration of crocin at 50 mg/kg and 100 mg/kg (55.1 and 68.1%, respectively). Metacestode pathology showed structural disruption of the germinal and laminated layers after crocin treatment. The crocin- and ABZ-treated groups presented significant increases in the levels of interleukin (IL)-2 and IL-4. Furthermore, crocin inhibited the expression of matrix metalloproteinases (MMPs) (MMP2 and MMP9) and promoted collagen deposition in the metacestode.

**Conclusions:**

Crocin was demonstrated to exert parasiticidal activity against *E. multilocularis* in vitro and in vivo, and can be developed as a novel drug for the treatment of AE.

**Graphical abstract:**

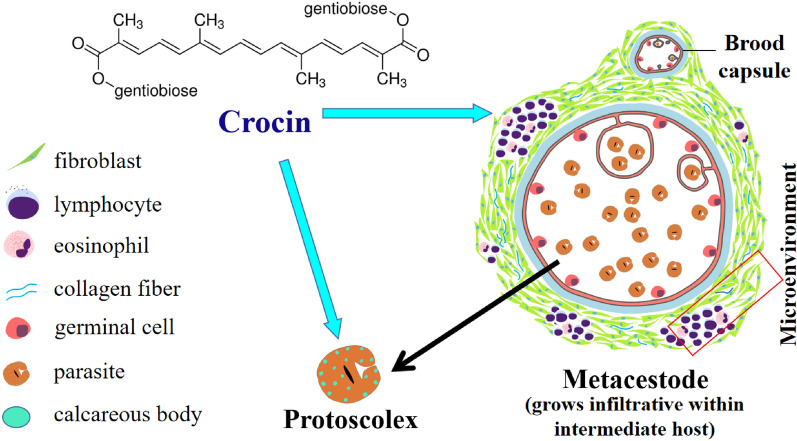

**Supplementary information:**

The online version contains supplementary material available at 10.1186/s13071-021-04866-4.

## Background

Alveolar echinococcosis (AE) is a rare zoonotic parasitic disease in humans caused by the larvae of *Echinococcus multilocularis*. The larvae grow in a malignant tumour-like pattern and can spread to the lungs, brain, kidneys and other distant organs through the blood circulatory system [1, 2]. *E. multilocularis* is largely restricted to the northern hemisphere, and the highest prevalence rates of infection occur in Central Asia, Russia, northwestern China, and parts of Europe and Japan [3]. The life cycle of *Echinococcus* involves two mammalian hosts; carnivores are usually the final hosts, while herbivores are the intermediate hosts [4]. Humans are accidental intermediate hosts and are usually infected via the consumption of food or water contaminated by eggs or by direct contact with infected soil or dogs. Oncospheres pass through the portal venous system and reach the liver, where they usually settle and develop as larvae (metacestodes) [5].

The incubation period of AE infection is approximately 5–15 years, and there are no early clinical symptoms. Once patients are diagnosed with AE, the disease is usually at an advanced stage and the patients have obvious symptoms and signs, including abdominal pain, jaundice, weight loss and even liver failure [6]. Metacestode tissues can proliferate indefinitely via exogenous buds and invade adjacent tissues, making the lesions resemble tumours. AE is considered to be the most lethal helminthosis, and the mortality rate in untreated or inadequately treated AE patients is > 90% within 10–15 years of diagnosis [5]. Surgery is the treatment of choice when the lesions can be completely removed [6]. In addition, all patients should receive chemotherapy with benzimidazoles, mainly albendazole (ABZ) and mebendazole (MBZ). However, benzimidazoles are parasitostatic, necessitating long-term treatment [7]. Therefore, new chemotherapies are needed to overcome these deficiencies and provide an alternative to conventional drug therapy for AE.

Multiple alternative compounds for the treatment of AE have been analysed in vitro using cultured metacestode vesicles and in vivo using rodent models, including other benzimidazole derivatives, broad-spectrum anti-infective drugs and anticancer drugs [8]. Notably, medicinal plants have gradually begun to be used to treat many diseases, including parasitosis [9]. The medicinal compounds in these plants have strong pharmacological activity and low toxicity [9].

Crocin is a water-soluble carotenoid found in the traditional Chinese medicinal plant *Crocus sativus*. Crocin has been proven to exert antitumour [10], antiangiogenic [11] and anti-inflammatory effects [12], and can promote apoptosis [13], protect the liver and gallbladder [14], and help ameliorate diabetes [15]. A previous study showed that crocin exerts antibacterial and antiparasitic effects [16]. Crocin plays an antitumour role mainly by inhibiting the expression of matrix metalloproteinases (MMPs) [17], inhibiting cancer cell invasion and promoting cancer cell apoptosis. Crocin is a potent antioxidant with the ability to decrease lipid peroxidation and mitochondrial and lysosomal membrane damage and thus protect the liver [18]. However, the effects of crocin on echinococcosis are unknown.

In this study, we evaluated the in vitro anthelmintic effects of crocin on metacestodes, germinal cells and protoscoleces of *E. multilocularis*. Furthermore, the in vivo efficacy and cytotoxicity of crocin were investigated in experimentally infected mice.

## Methods

### Experimental animals

Specific pathogen-free male BALB/c mice (aged 4 weeks, 16–18 g) were purchased from Qinglongshan Animal Breeding Farm, Jiangning District, Nanjing (no. 201901649). The mice were housed in a specific pathogen-free environment at a room temperature of 25 °C under a 12-h/12-h light/dark cycle. The mice were given free access to food and water. *E. multilocularis* protoscoleces were obtained from Mongolian gerbils preserved in our laboratory.

### Cells and chemicals

Human foreskin fibroblasts (HFFs) and Reuber rat hepatoma (RH) cells were purchased from Procell (Wuhan, China). Dulbecco’s modified Eagle medium (DMEM), Medium 199 and foetal bovine serum (FBS) were obtained from Gibco (Auckland, New Zealand). Solutions containing trypsin–ethylenedinitrilotetraacetic acid, penicillin/streptomycin (100×) and gentamicin were purchased from Procell. Crocin and albendazole sulphoxide (ABZSO) were purchased from Yuanye Bio-Technology (Shanghai, China). Crocin was prepared as 10-mM stocks in double-distilled water or culture medium and stored at − 20 °C.

### In vitro cultivation of *E. multilocularis* metacestodes

Larval material was obtained from euthanized BALB/c mice experimentally infected with homogenized larval tissue of *E. multilocularis*, which was originally isolated from a naturally infected plateau pika (*Ochotona curzoniae*) collected in Yushu, Qinghai Province, China. Molecular identification of the isolate of *E. multilocularis* was performed as described by Nitta et al. [19]. *E. multilocularis* metacestodes were prepared and cultured as previously described [20]. Briefly, the metacestodes isolated from experimentally infected BALB/c mice were crushed through a metal tea strainer and incubated overnight in phosphate-buffered saline (PBS) containing 1% penicillin/streptomycin. Then, 1 mL of metacestode tissue was cocultured with 5 × 10^6^ RH feeder cells in DMEM containing 10% FBS and 1% penicillin/streptomycin at 37 °C and 5% CO_2_ with medium changes once a week. The RH cells were grown to confluence and then trypsinized and diluted 1:20 in fresh culture medium once a week. In vitro cultured metacestode vesicles were used for experiments when they reached diameters of 2–4 mm [20]. Some of the metacestode vesicles were fixed with 4% paraformaldehyde for pathological examination.

### Confirmation of metacestode vesicles by real-time polymerase chain reaction

Metacestode vesicles (without host cell contamination) were detected by real-time polymerase chain reaction (RT-PCR). Total RNA was extracted from metacestode vesicles and host tissue (normal mouse livers) using an RNAsimple Kit (TianGen Biotech, Beijing, China) according to the manufacturer’s instructions. First-strand complementary DNA (cDNA) was synthesized using a FastKing gDNA Dispelling RT SuperMix Kit (TianGen Biotech). PCR was performed using specific primers for amplification of *E. multilocularis* glyceraldehyde-3-phosphate dehydrogenase (GAPDH) [19] and mouse GAPDH (forward, 5′-CGTGGGGCAGCCCAGAACAT-3′; reverse, 5′-GAGCAATGCCAGCCCCAGCA-3′).

RT-PCR was performed using a MasterCycler Pro S (Applied Biosystems, Foster City, CA) in a final volume of 20 μL, including 10 μL of 2 × Taq PCR MasterMix (TianGen Biotech), 0.6 μL of each primer (10 μM), 2 μL of cDNA product, and 6.8 μL of double-distilled water. Amplification was performed using the following conditions: 3 min at 94 °C followed by 30 cycles of 94 °C for 30 s, 60 °C for 30 s and 72 °C for 30 s, and a final extension step at 72 °C for 3 min. The PCR products were separated by 2% agarose gel electrophoresis and stained with GeneRed (TianGen Biotech) for visualization under ultraviolet light. Metacestode germinal cells were subsequently identified in the same manner.

### In vitro assessment of crocin activity against *E. multilocularis* metacestodes

To evaluate the efficacy of crocin against *E. multilocularis* metacestodes, a phosphoglucose isomerase (PGI) assay was performed in which the release of the enzyme PGI was examined upon the physical impairment of the metacestodes [20]. In brief, medium without phenol red (DMEM; 1% penicillin/streptomycin sulfate, 2 mM l-glutamine) was added to the same volume of vesicles, and then, suspended vesicles were distributed into 48-well plates (12–15 vesicles per well). Subsequently, the metacestodes were incubated for 5 days with different concentrations (0, 0.5, 1.0, 2.5, 5, 10, 20, 40, 80, 120 and 160 μM) of crocin, after which PGI release was quantified exactly as stated by Stadelmann et al. [21]. Triton X-100 (0.1% in PBS) was applied as a positive control (to induce maximal release of vesicle fluid). Each condition was tested in biological triplicate. After 5 days of incubation, 200 μL of medium supernatant was collected from each well and stored at − 20 °C until further measurements were performed. PGI measurements were performed as described previously [20], except that an Infinite M200 Pro reader (Tecan, Männedorf, Switzerland) was used to measure the increase in absorbance at 340 nm. PGI activity was calculated in Microsoft Office Excel 2010 from the linear regression of the enzyme reaction over time, and is presented as a percentage relative to the values obtained after treatment of vesicles with 0.1% Triton X-100.

### Assessment of in vitro toxicity in HFFs and RH cells

An alamarBlue assay was used to assess the toxicity of crocin to confluent and preconfluent mammalian cells in vitro [22]. HFFs and RH cells were seeded into 96-well cell culture plates in DMEM supplemented with 10% FBS and 1% penicillin/streptomycin at 37 °C and 5% CO_2_. To detect the growth-inhibiting effects on confluent cells, HFFs and RH cells were seeded at densities of 10,000 cells per well and 50,000 cells per well, respectively. After overnight culture, crocin was added and diluted in serial 1:10 serial dilutions down to 100 μM. To detect growth-inhibiting effects on proliferating cells, HFFs and RH cells were seeded at densities of 1000 and 5000 cells per well, respectively. Crocin was added after 6 h of cell attachment. After treatment for 5 days, cell viability was measured using an alamarBlue assay. The cells were washed three times in PBS, and resazurin was added to 10 mg/L. Fluorescence at 595 nm was measured at 0 and 4 h with an Infinite M200 Pro reader (Tecan). The values obtained at 0 h were subtracted from those obtained at 4 h. IC50 values were calculated using an online IC50 calculator (https://www.aatbio.com/tools/ic50-calculator) after logit-log transformation, and averages and SDs of six independent setups were calculated.

### Assessment of in vitro toxicity in *E. multilocularis* germinal cells

To evaluate the activity of crocin against parasitic stem cells, germinal cells were obtained from in vitro cultured metacestode vesicles as described by Spiliotis et al., with some modifications [23]. Briefly, 20 units of cells were distributed amongst black 384-well plates. Different concentrations of crocin (0, 0.5, 1.0, 2.5, 5, 10, 20, 40, 80 and 160 μM) were added to the cells. After culture at 37 °C for 5 days under a humid nitrogen atmosphere, 25 μL of CellTiter-Glo containing 1% Triton X-100 was added. The plates were incubated at room temperature and in the dark for 15 min. After the total destruction of the cellular aggregates, luminescence was measured using an Infinite M200 Pro reader (Tecan). The values for 0 μM were set to 100% viability. IC50 values were calculated using an online IC50 calculator (https://www.aatbio.com/tools/ic50-calculator) after logit-log transformation. Four independent replicates were conducted.

### Isolation of *E. multilocularis* protoscoleces

*E. multilocularis* protoscoleces were obtained from Mongolian gerbils maintained in our laboratory. Briefly, metacestode tissues were isolated from the abdominal cavities of Mongolian gerbils after euthanasia with CO_2_. The metacestode tissues were minced in precooled 0.9% normal saline (NS), and *E. multilocularis* protoscoleces were filtered using four-layer sterile gauze into a 50-mL sterile centrifuge tube. The initial filtrate was then filtered again using a 100-μm cell strainer, and the resulting filtrate was further filtered with a 40-μm cell strainer for the removal of calcareous bodies. During the filtration process, the *E. multilocularis* protoscoleces were constantly washed in precooled 0.9% NS. Finally, *E. multilocularis* protoscoleces were allowed to naturally settle to the bottom of the container and washed using precooled NS ten times. Each mouse was inoculated intraperitoneally with 3000 protoscoleces.

### In vitro effects of crocin on *E. multilocularis* protoscoleces

Viable protoscoleces were cultured in Medium 199 (Gibco) supplemented with 1% penicillin/streptomycin, 50 μg/mL gentamicin and 4 mg/mL glucose according to a previous method [24]. Protoscoleces were transferred to 24-well cell culture plates and incubated with different thiacloprid concentrations (0, 0.5, 1.0, 2.5, 5, 10, 20, 40, 60, 80 and 160 μM). ABZSO (15 μM) was used as a positive control [25]. The protoscoleces were cultured in an incubator at 37 °C and 5% CO_2_ for 7 days. Protoscoleces were observed each day in triplicate with an optical microscope (BX51; Olympus, Tokyo, Japan). We determined the viability percentages by calculating the percentage of dead and live protoscoleces among 300 protoscoleces by means of an eosin exclusion experiment [26]. Live protoscoleces do not absorb eosin stain; however, in dead protoscoleces, eosin enters the cell, and the protoscoleces become red. To reduce the bias as much as possible, protoscolex viability was observed by two experimenters under double-blind conditions. Each experiment was repeated three times.

### Scanning electron microscopy and transmission electron microscopy

*E. multilocularis* protoscoleces or metacestode vesicles were treated with crocin as described above. The protoscoleces or metacestode vesicles were fixed in 2.5% glutaraldehyde buffer (pH = 7.25) at 4 °C for 2 h. Then, the samples were postfixed in 1 mL of osmium tetroxide (2% in 0.1 M cacodylate buffer) for 2 h, washed three times in water, and dehydrated in increasing concentrations of ethanol (30%, 50%, 70%, 80%, 90%, 95% and 2× 100%). Subsequently, the samples were incubated in hexamethyl disilazine for 2 min. After evaporation at room temperature, the samples were sputter-coated with gold and inspected in a Hitachi SU8100 or SU8020 scanning electron microscope operating at 3.0 kV. For transmission electron microscopy (TEM) analysis, dehydrated samples were embedded in Epon 812 resin with three subsequent resin changes for 2 days and incubated at 65 °C overnight for polymerization. Sections (50 nm) were prepared using an ultramicrotome (Leica, Wetzlar, Germany) and loaded onto Formvar carbon-coated nickel grids (Gilder Grids, Grantham, Lincolnshire, UK). Finally, the samples were stained with uranium acetate and lead citrate and observed using a JEM-1400plus transmission electron microscope (JEOL, Tokyo, Japan).

### Determination of the in vivo toxicity of crocin

To assess the subacute toxicity of crocin in vivo, mice were randomly assigned to the control group (orally administered NS, *n* = 6), the group orally administered crocin at 50 mg/kg (*n* = 6) and the group orally administered crocin at 100 mg/kg (*n* = 6). The treatment was conducted for 6 weeks. At the end of the experiment, blood samples were collected from the eyeballs of mice under anaesthesia before euthanasia and preserved in ethylenedinitrilotetraacetic acid-K2 anticoagulant tubes for white blood cell, haemoglobin and platelet analysis. For assessment of biochemical indicators, the blood samples were incubated in anticoagulant-free tubes at 37 °C for 1 h and then centrifuged at 3500 r.p.m. for 10 min. The isolated serum samples were used to detect the relative levels of alanine aminotransferase, aspartate aminotransferase, total bilirubin, direct bilirubin, indirect bilirubin, total protein, albumin, alkaline phosphatase, creatinine and blood urea nitrogen. Subsequently, the mice were sacrificed, and their livers and kidneys were harvested, fixed in 4% paraformaldehyde, prepared for haematoxylin–eosin (HE) staining and observed under a BX51 microscope (Olympus).

### Determination of the in vivo effects of crocin on *E. multilocularis* metacestodes

BALB/c mice were infected with *E. multilocularis* protoscoleces for 8 weeks. Later, the mice were randomly distributed into four groups as follows: the control group (orally administered 0.4 mL of NS, *n* = 10); the ABZ group (orally administered 200 mg/kg ABZ dissolved in 0.4 mL of NS, *n* = 10); the crocin50 group (orally administered 50 μg/mL crocin dissolved in 0.4 mL of NS, *n* = 10); and the crocin100 group (orally administered 100 μg/mL crocin dissolved in 0.4 mL of NS, *n* = 10). Uninfected mice were used as a blank group. Drug administration was performed once a day at a fixed time point. After 6 weeks of treatment, blood samples were collected from the eyeballs before CO_2_ euthanasia. Blood samples were incubated at 37 °C for 1 h and centrifuged at 3500 r.p.m. for 10 min at 4 °C. The serum was harvested and stored at − 20 °C for interleukin (IL)-2, IL-4 and immunoglobulin E (IgE) detection. In addition, metacestodes in the abdominal cavity were carefully harvested and weighed. The host tissue surrounding the metacestode was isolated under a stereomicroscope (SZ51; Olympus) and stored at -80 °C for further experiments. Some metacestode tissues were fixed in 4% paraformaldehyde for pathological examinations, and some were used for TEM analysis after fixation in 2.5% glutaraldehyde buffer and staining.

### HE staining

Tissues from experimental animals were washed in PBS and fixed in 4% paraformaldehyde at room temperature for 36 h. The next day, the samples were dehydrated, embedded, and sliced into 5-μm sections. After dewaxing and dehydration, the sections were stained with haematoxylin for 2–5 min and with eosin for 15 s. The sections were sealed with neutral gum and observed under a light microscope (BX51; Olympus).

### Periodic acid Schiff and Masson trichrome staining

Metacestode tissues were fixed in paraformaldehyde, embedded in paraffin, sectioned at 5 μm, and stained with periodic acid Schiff (PAS; Solarbio, Beijing, China) and Masson trichrome (Solarbio) according to the instructions for evaluation of fibrosis and the extent of extracellular matrix deposition in host tissues around metacestodes. PAS-positive substances appear red or purplish red. The collagen fibres were blue or green after Masson trichrome staining. The sections were finally observed under a microscope (BX51; Olympus).

### Enzyme-linked immunosorbent assay

The serum levels of IL-2, IL-4 and IgE in experimental animals were calculated against standard curves using commercial enzyme-linked immunosorbent assay (ELISA) kits (Elabscience Biotechnology, Wuhan, China). The optical density value at 450 nm was read with an Infinite M200 Pro enzyme reader (Tecan), and the concentration was calculated.

### Real-time quantitative PCR

Total RNA was isolated from host tissues around the metacestodes using TRIzol (Thermo Scientific, Waltham, MA), and the integrity of the RNA was assessed by examination of the 18S and 28S fragments of ribosomal RNA following agarose gel electrophoresis. Later, the RNA purity and concentration were determined using a NanoDrop 2000 spectrophotometer (NanoDrop Technologies, Wilmington, DE). Qualified RNA (2 μg) was reverse-transcribed into cDNA using FastKing gDNA Dispelling RT SuperMix (TianGen Biotech), which was sub-packaged and preserved at -80 °C for use. The primer sequences were as follows: MMP2, forward 5′‐TTGGGCTGCCCCAGACAGGT‐3′, reverse 5′‐GTCCCACTTGGGCTTGCGGG‐3′; MMP9, forward 5′‐AGCCCCTGCTCCTGGCTCTC‐3′, reverse 5′‐CTGCCAGCTGGGTGTCCGTG‐3′; collagen I, forward 5′‐TGGCCAGATGGGTCCCCGAG‐3′, reverse 5′‐AGGGGGTCCAGCAGCACCAA‐3′; collagen III, forward 5′‐ACCTGCAGGACCCACTGGCA‐3′, reverse 5′‐GACCACGCCCACCGGGAAAG‐3′; and RPS18, forward 5′‐GCCAGGTTCTGGCCAACGGT‐3′, reverse 5′‐CCCTGCGGCCAGTGGTCTTG‐3′. The reaction buffers for RT-qPCR were prepared using a commercial kit (TianGen Biotech), and RT-qPCR was conducted using an ABI Q5 RT-qPCR system (Applied Biosystems). The PCR thermal cycling protocol applied consisted of one step of 2 min at 50 °C for pretreatment and one step of 10 min at 95 °C for initial denaturation followed by 40 cycles consisting of a denaturation step for 15 s at 95 °C and an annealing step/extension step for 30 s at 60 °C. A melting curve analysis was performed after the final amplification period with a temperature gradient of 95 °C for 15 s, 60 °C for 15 s, and 95 °C for 15 s. The relative messenger RNA (mRNA) levels were calculated using the 2^−△△CT^ method [27].

### Western blotting analysis

Host tissues from around metacestodes were lysed on ice using radioimmunoprecipitation assay buffer (Thermo Scientific) containing phenylmethylsulfonyl fluoride, and the lysates were centrifuged at 4 °C and 12,000 r.p.m. for 10 min. The supernatant was collected, diluted in 5× loading buffer and boiled in a 95 °C water bath for 10 min. The prepared protein samples were preserved at − 80 °C after measurement of the protein concentration. The protein samples were subjected to sodium dodecyl sulfate–polyacrylamide gel electrophoresis at 20 μg per lane and transferred to 0.2-μm polyvinylidene fluoride membranes (Merck Millipore, Darmstadt, Germany). Nonspecific antigens on the membrane were blocked with Tris-buffered saline 0.05%—Tween 20 containing 5% nonfat milk at room temperature for 1 h. The polyvinylidene fluoride membranes were subjected to immunoblotting with primary antibodies against MMP2 (1:1000; Abclonal, Wuhan, China), MMP9 (1:1000; Abclonal) and β-actin (1:1000; Abclonal) at 4 °C overnight. The next day, they were washed and incubated with horseradish peroxidase-labelled goat anti-rabbit IgG (1:5000) at room temperature for 1 h. After washing, an enhanced chemiluminescence (Thermo Scientific) method was used to expose the bands, and the greyscale values were normalized to that of β-actin.

### Statistical analysis

The data are expressed as the mean ± SD and were plotted with GraphPad Prism 8.0 software. Multiple comparisons between more than two groups were analysed using one-way ANOVA or the Kruskal–Wallis test (nonparametric). Median inhibitory concentrations (IC50) and median effective concentrations (EC50) were calculated using an online half-max graphing calculator (https://www.aatbio.com/index.html). A value of *P* < 0.05 was considered significant.

## Results

### In vitro activity of crocin against *E. multilocularis* metacestodes

**Fig. 1:**
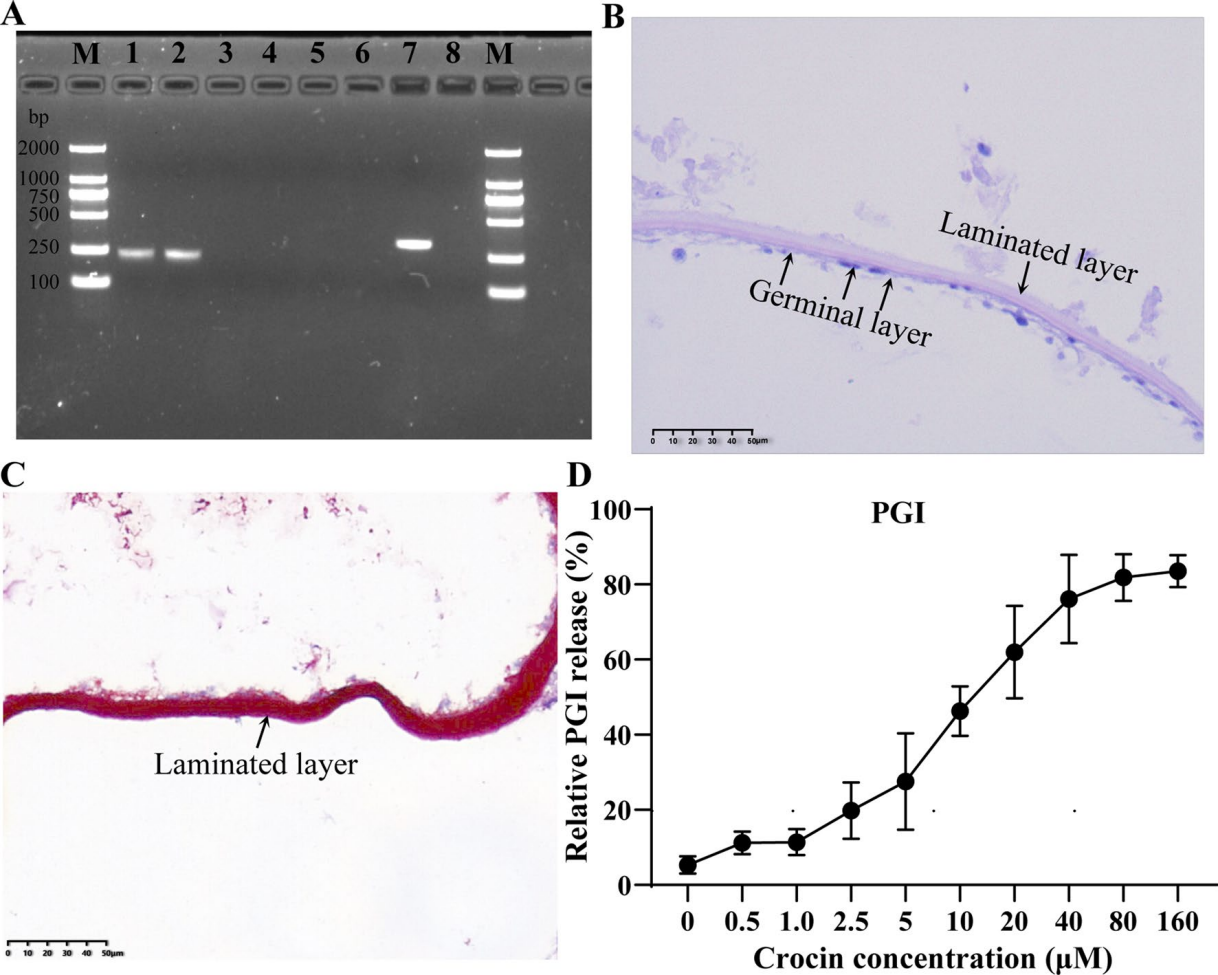
**a**–**d** Effects of crocin against *Echinococcus multilocularis* metacestodes in vitro. **a** Identification of metacestodes and germinal cells. *E. multilocularis* glyceraldehyde-3-phosphate dehydrogenase (GAPDH) and mouse GAPDH were used for the characterization of *E. multilocularis* metacestodes and germinal cells (without host cell contamination).* Lanes* 1,* 5* Metacestodes;* lanes 2*,* 6* germinal cells;* lanes 3*,* 7* mouse liver;* lanes 4*,* 8* negative control. **b** Pathological observation of metacestode vesicles. An obvious germinal layer (GL) and a laminated layer (LL) were visible in metacestode sections after haematoxylin–eosin (HE) staining.* Scale bar* = 50 μm. **c** Metacestode sections were stained with periodic acid Schiff (PAS). The sections presented a strongly PAS-positive basophilic LL.* Scale bar* = 50 μm. **d** Effects of different concentrations of crocin against *E. multilocularis* metacestodes. The effects of a series of concentrations ranging from 0.5 to 160 μM on* E. multilocularis* metacestodes were tested by phosphoglucose isomerase (*PGI*) assay in triplicate. Triton X-100 (0.1%) served as a positive control; the result for the positive control group was set to 100%. The data are presented as the mean ± SD

In the RT-PCR analysis, mouse GAPDH was not amplified from metacestode vesicles or germinal cells, thus confirming the absence of host cells, while the *E. multilocularis* GAPDH-specific gene was amplified from metacestode vesicles and germinal cells (Fig. 1a). HE staining revealed an obvious germinal layer in metacestode vesicles (Fig. 1b). A PAS-positive laminated layer was observed (Fig. 1c). The in vitro activity of crocin against *E. multilocularis* metacestodes was assessed using the PGI assay (Fig. 1d). The enzymatic activity is expressed relative to the PGI activity obtained from vesicles permeabilised with Triton X-100. Crocin showed significant anti-metacestode activity, and the EC50 value was determined to be 11.36 ± 4.06 μM by the PGI assay.

### Crocin cytotoxicity towards HFFs and RH cells

**Fig. 2:**
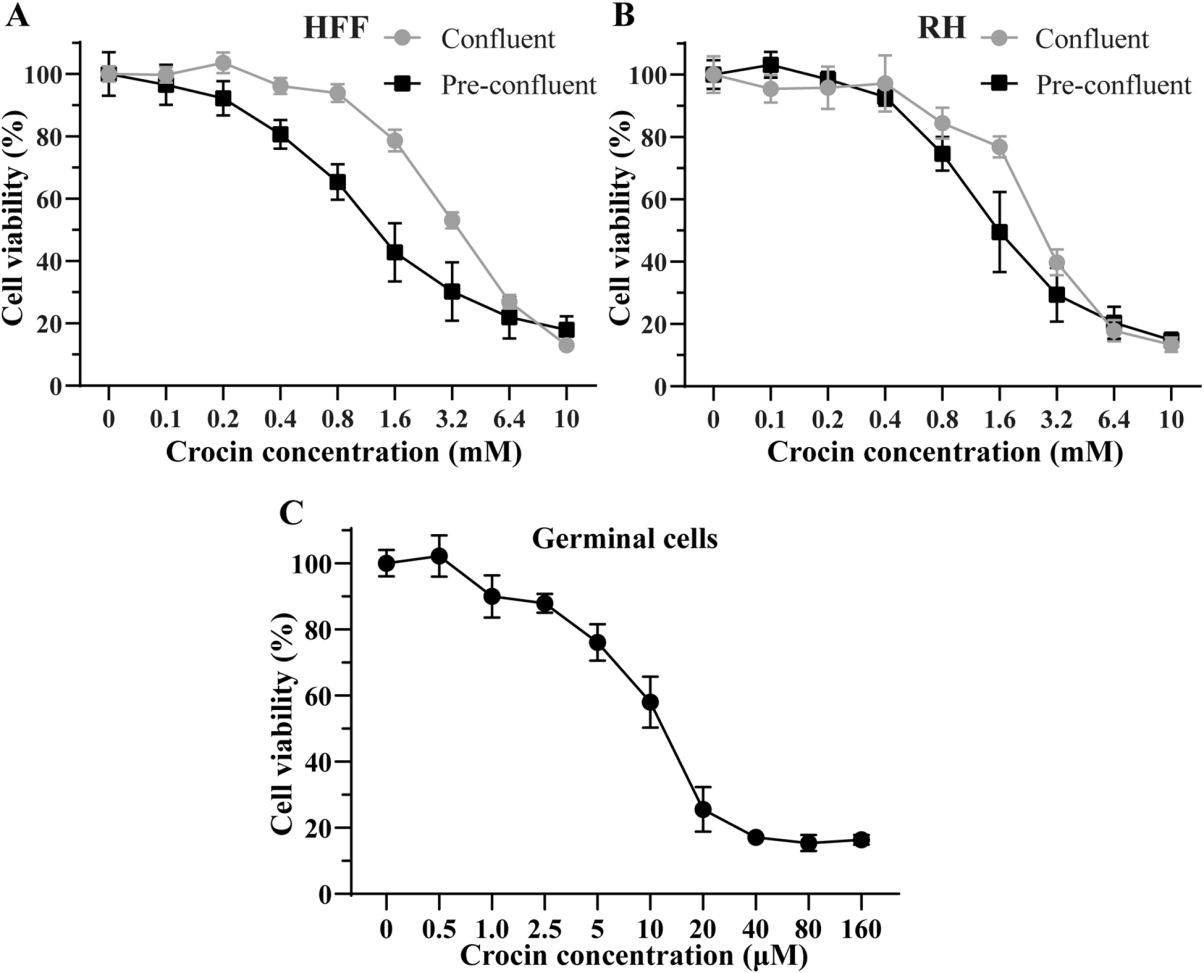
**a**–**c** Toxicity of crocin towards mammalian cells and germinal cells. In vitro activity of crocin against human foreskin fibroblast (*HFFs*) (**a**), Reuber rat hepatoma (*RH*) cells, (**b**) and germinal cells (**c**). HFF and RH cell viability was tested via alamarBlue assay, while *E. multilocularis* germinal cell viability was assessed via CellTiter-Glo assay. The data are presented as the mean ± SD

The cytotoxicity of crocin was assessed by an alamarBlue assay using confluent and preconfluent cultures of HFFs and RH cells (Fig. 2a, b). The IC50 values of crocin for confluent and preconfluent HFF cells were 3.82 ± 0.82 mM and 1.09 ± 0.37 mM, respectively, while the IC50 values for confluent and preconfluent RH cells were 2.64 ± 0.37 mM and 1.43 ± 0.52 mM, respectively. The IC50 values of crocin for preconfluent cells were generally lower than those for confluent cells. In addition, crocin was less toxic to HFFs and RH cells than to *E. multilocularis* metacestodes.

### Effect of crocin on the viability of *E. multilocularis* germinal cells

A CellTiter-Glo assay was used to determine the effects of crocin on germinal cells (Fig. 2c). Crocin significantly inhibited germinal cell proliferation. Crocin at a concentration greater than 40 μM significantly reduced the number of viable cells, with an inhibition rate of more than 80% (Fig. 2c). At 10 and 20 μM, crocin inhibited germinal cell viability by 45.61 ± 5.53% and 64.32 ± 8.95%, respectively. The IC50 of crocin for germinal layer cells was 10.05 ± 2.23 μM.

### Electron microscopic visualization of the effects of crocin treatment in *E. multilocularis* metacestodes

**Fig. 3:**
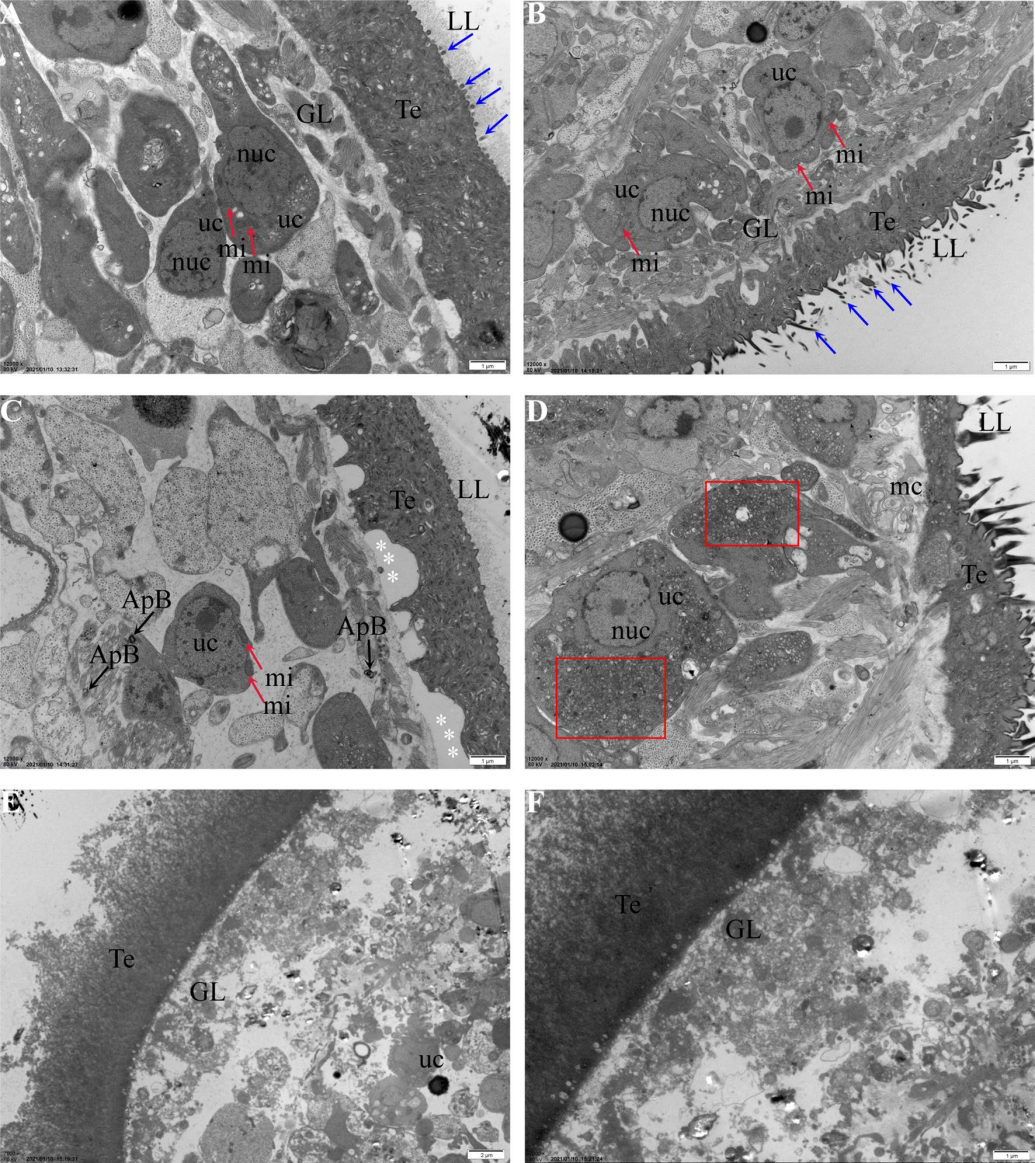
**a**–**f** Transmission electron microscopy (TEM) analysis of *E. multilocularis* metacestodes treated with crocin. **a** Normal metacestode structure. The outermost LL, tegument (*Te*) and inner GL are clearly discernible. Metacestode vesicles were cultured without crocin treatment for 5 days and processed for TEM.* Blue arrows* indicate microtriches that originate at the Te and protrude into the LL.* uc* Undifferentiated cells,* mi* mitochondria,* nuc* nucleus. **b** Metacestodes treated with 2.5 μM crocin. Microtriches (*blue arrows*) are still intact. Undifferentiated cells and mi exhibit similar morphology to the metacestode not treated with crocin. **c**, **d** Metacestodes treated with 5 μM crocin. The GL is separated from the Te (indicated by* white asterisks*). The cytoplasm of GL cells was filled with electron-dense deposits (marked by a* red box*), and vacuoles containing membrane stacks (*ms*) resembling autophagosomes (*ApB*) were visible. **e**, **f** Metacestodes treated with 10 μM crocin. The GL structure was obviously damaged. A few small undifferentiated cells were observed.** a**–**d**,** f** *Scale bar* = 1 μm; **e** *scale bar* = 2 μm. For other abbreviations, see Fig. 1

TEM images of metacestodes cultured for a period of 5 days without crocin are shown in Fig. 3a. The outer part of the parasite tissue is composed of laminated layers, which are rich in carbohydrates and separate the parasitic tissue from the surrounding host tissue. The inner surface of the laminated layer is the tegument, which features numerous microtriches that protrude into the laminated layer. The germinal layer, which adheres to the capsule, contains a variety of cell types, including muscle cells, sub-tegumentary cytons and undifferentiated stem cells, and connective tissue. Metacestodes treated with 0.25 μM crocin did not show obvious changes; a representative micrograph is shown in Fig. 3b. After metacestodes were treated with 5 μM crocin, the germinal layer was separated from the tegument, and more electron-densit bodies appeared in the cytoplasm of parasite stem cells (Fig. 3c, d). After 10 μM crocin treatment, the structure of the tegument and germinal layer was obviously destroyed (Fig. 3e, f).

### Effects of crocin on *E. multilocularis* protoscoleces

**Fig. 4:**
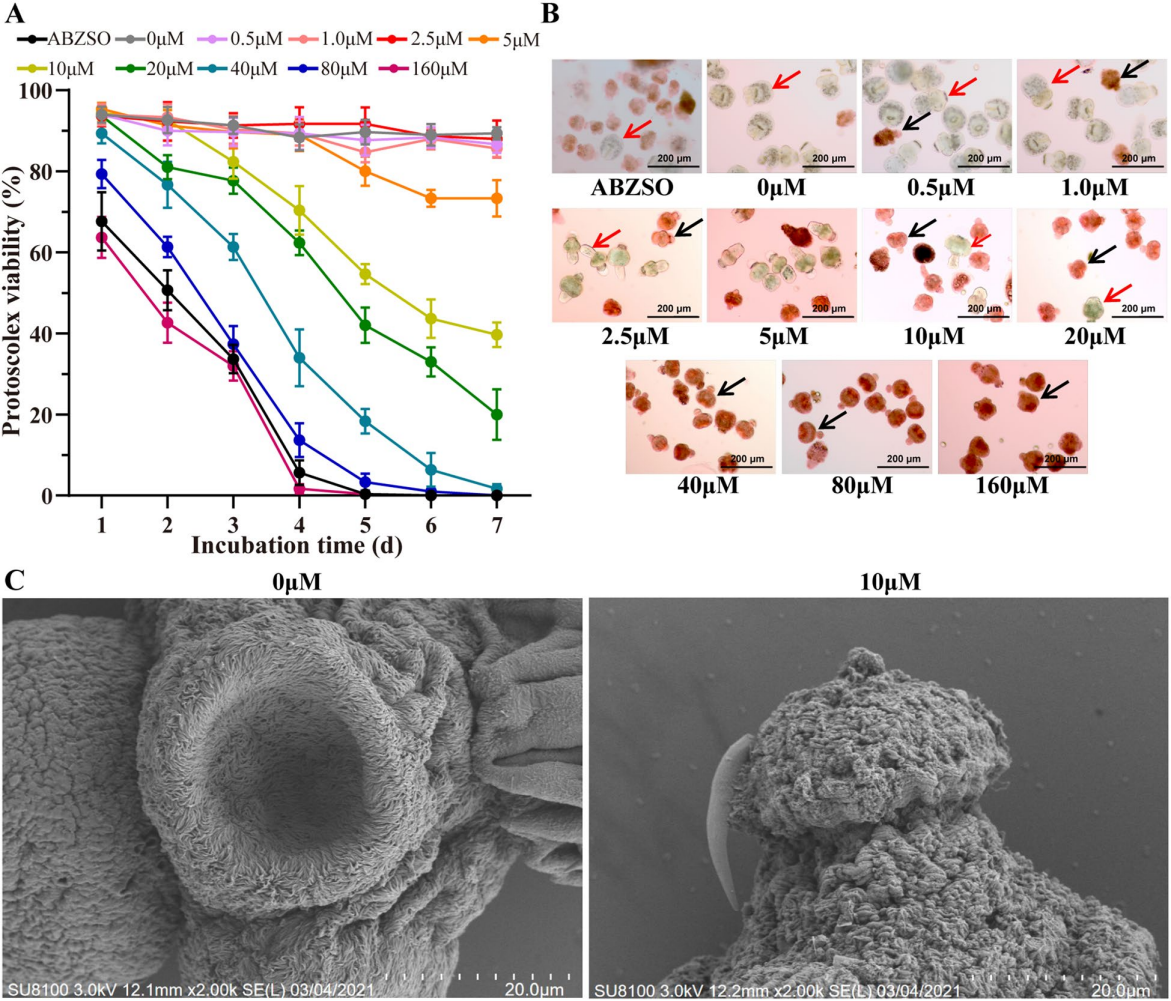
**a**–**c** Effects of crocin against *E. multilocularis* protoscoleces. **a** Viability of protoscoleces cultured in vitro. The protoscoleces were exposed to crocin for 7 days. To evaluate the survival of protoscoleces, a 0.1% eosin staining exclusion method was used daily. The positive control treatment was 15 μM albendazole sulphoxide (*ABZSO*). The data are presented as the mean ± SD obtained from three independent experiments. **b** Effects of crocin treatments on morphology and structural integrity. After treatment with crocin for 5 days, eosin staining was used to observe the protoscoleces.* Scale bar* = 200 μM. **c** Scanning electron microscopy analysis of *E. multilocularis* protoscoleces exposed to 10 μM crocin for 5 days.* Scale bar* = 20 μM

The killing effect of crocin on *E. multilocularis* protoscoleces in vitro was further assessed. The dead protoscoleces were distinguished from the viable protoscoleces by eosin exclusion and were observed under a light microscope. The survival of *E. multilocularis* protoscoleces after exposure to different concentrations of crocin is shown in Fig. 4a. Without crocin treatment, more than 85% of protoscoleces remained viable after 7 days of incubation, and no changes in structure or ultrastructure were observed throughout the experimental period (Fig. 4b). Crocin treatment had a protoscolicidal effect. The survival rate of protoscoleces was less than 20% after 4 days of treatment with 80 μM crocin. After 10-μM crocin treatment for 7 days, the survival rate of protoscoleces was less than 40%. At 5 μM, crocin showed no obvious killing effect on protoscoleces. The effects of crocin on the *E. multilocularis* protoscolex ultrastructure were visualized by scanning electron microscopy (SEM) after 7 days of incubation (Fig. 4c). After 10-μM crocin treatment, the protoscoleces were significantly contracted, and the hooks had fallen off.

### In vivo toxicity of crocin

**Fig. 5:**
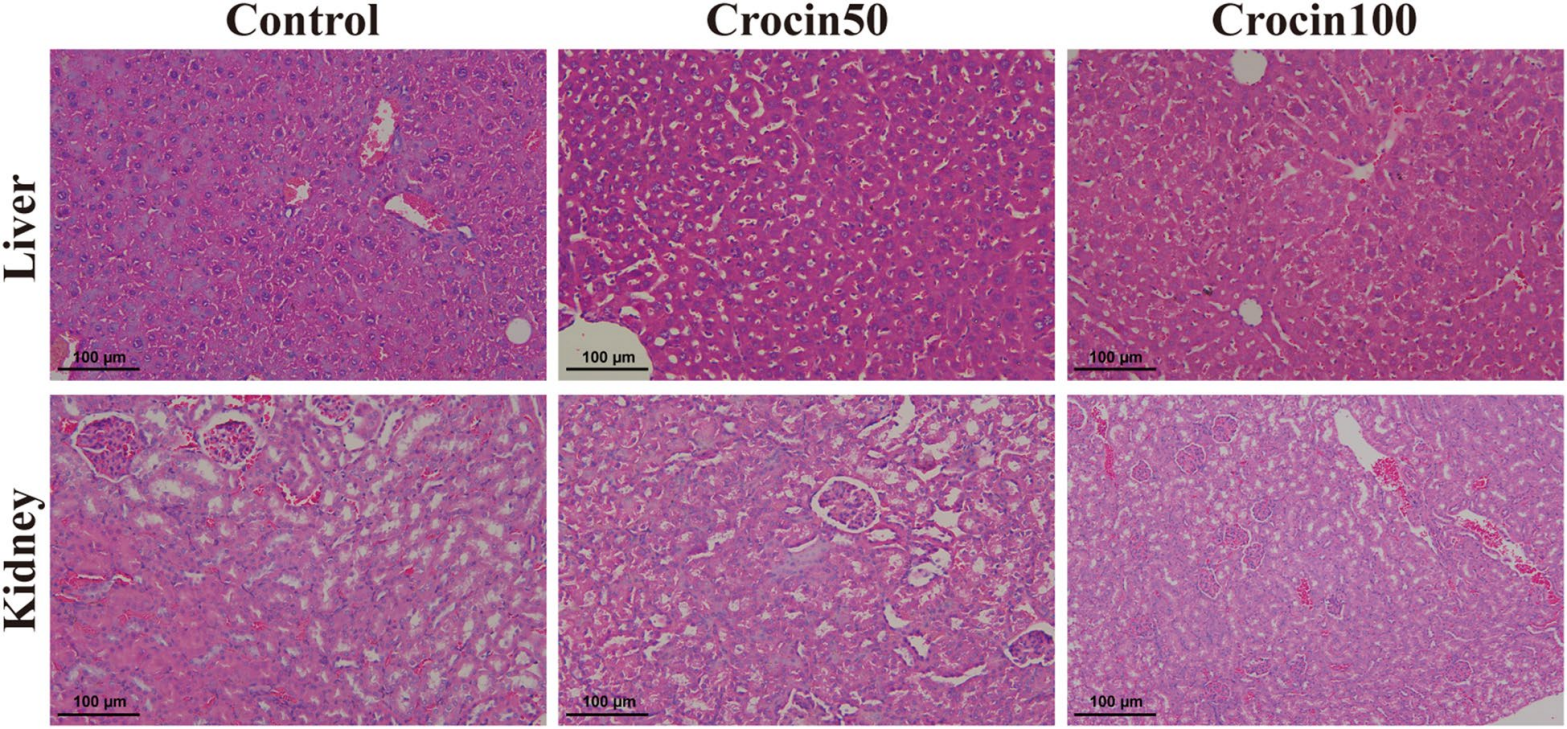
Evaluation of crocin toxicity in vivo. The mice were treated with oral crocin for 6 weeks and then sacrificed. Liver and kidney tissues were observed after HE staining.* Crocin50* Crocin administered at 50 mg/kg, *Crocin 100* crocin administered at 100 mg/kg

Adverse reactions to a drug can limit its clinical application to a large extent. Thus, we further analysed the in vivo toxicity of crocin by treating BALB/c mice with crocin for 6 weeks. The serum alanine aminotransferase, aspartate aminotransferase, total bilirubin, direct bilirubin, indirect bilirubin, total protein, albumin, alkaline phosphatase, creatinine and blood urea nitrogen levels and the blood white blood cell counts, haemoglobin levels and platelet counts are shown in Table 1. There were no significant differences in these biochemical and haematological parameters between the crocin-treated group and the control group. These tests showed that crocin did not significantly affect liver or kidney function in mice. Furthermore, the livers and kidneys of mice were subjected to histopathological examination after HE staining (Fig. 5). The livers and kidneys of mice treated with crocin did not show obvious histopathological changes or injuries.

**Table 1:** Blood cell indexes and serum biochemical indexes of BALB/c mice after 6 weeks of crocin treatment (*n* = 6)

BALB/c mice	Control	Crocin50	Crocin100	*F*	*P*
ALT (U/L)	62.17 ± 5.71	62.17 ± 3.19	65.50 ± 4.93	0.9945	0.3930
AST (U/L)	189.80 ± 25.90	186.30 ± 2.62	185.00 ± 22.26	0.0668	0.9356
TBIL (μmol/L)	0.66 ± 0.21	0.60 ± 0.10	0.59 ± 0.16	0.2914	0.7513
DBIL (μmol/L)	0.09 ± 0.04	0.13 ± 0.05	0.13 ± 0.04	1.7910	0.2008
IBIL (μmol/L)	0.55 ± 0.18	0.53 ± 0.15	0.60 ± 0.21	0.2591	0.7752
TP (g/L)	61.93 ± 2.23	62.30 ± 3.30	61.22 ± 3.21	0.2090	0.8137
ALB (g/L)	28.02 ± 1.95	29.08 ± 2.03	28.58 ± 1.66	0.4805	0.6277
ALP (U/L)	84.50 ± 4.64	83.67 ± 6.15	86.33 ± 8.02	0.2710	0.7663
CRE (μmol/L)	15.80 ± 3.06	16.03 ± 1.94	16.00 ± 2.97	0.0131	0.9870
BUN (mmol/L)	9.24 ± 0.95	7.11 ± 1.81*	7.30 ± 0.55*	5.5770	0.0155
White blood cells (× 10^9^/L)	8.14 ± 1.40	8.10 ± 0.99	8.08 ± 1.22	0.0040	0.9962
Haemoglobin (g/L)	129.30 ± 8.07	128.80 ± 9.50	129.30 ± 11.98	0.0050	0.9950
Platelets (× 10^9^/L)	466.20 ± 45.31	481.80 ± 64.36	466.80 ± 63.64	0.1379	0.8723

*Crocin50* Crocin administered at 50 mg/kg, *Crocin100* crocin administered at 100 mg/kg, *ALT* alanine aminotransferase, *AST* aspartate aminotransferase, *TBIL* total bilirubin, *DBIL* direct bilirubin, *IBIL* indirect bilirubin, *TP* total protein,*ALB* albumin, *ALP* alkaline phosphatase, *CRE* creatinine, *BUN* blood urea nitrogen

* *P* < 0.05 versus control

### Effects of crocin on *E. multilocularis* metacestodes in vivo

**Fig. 6:**
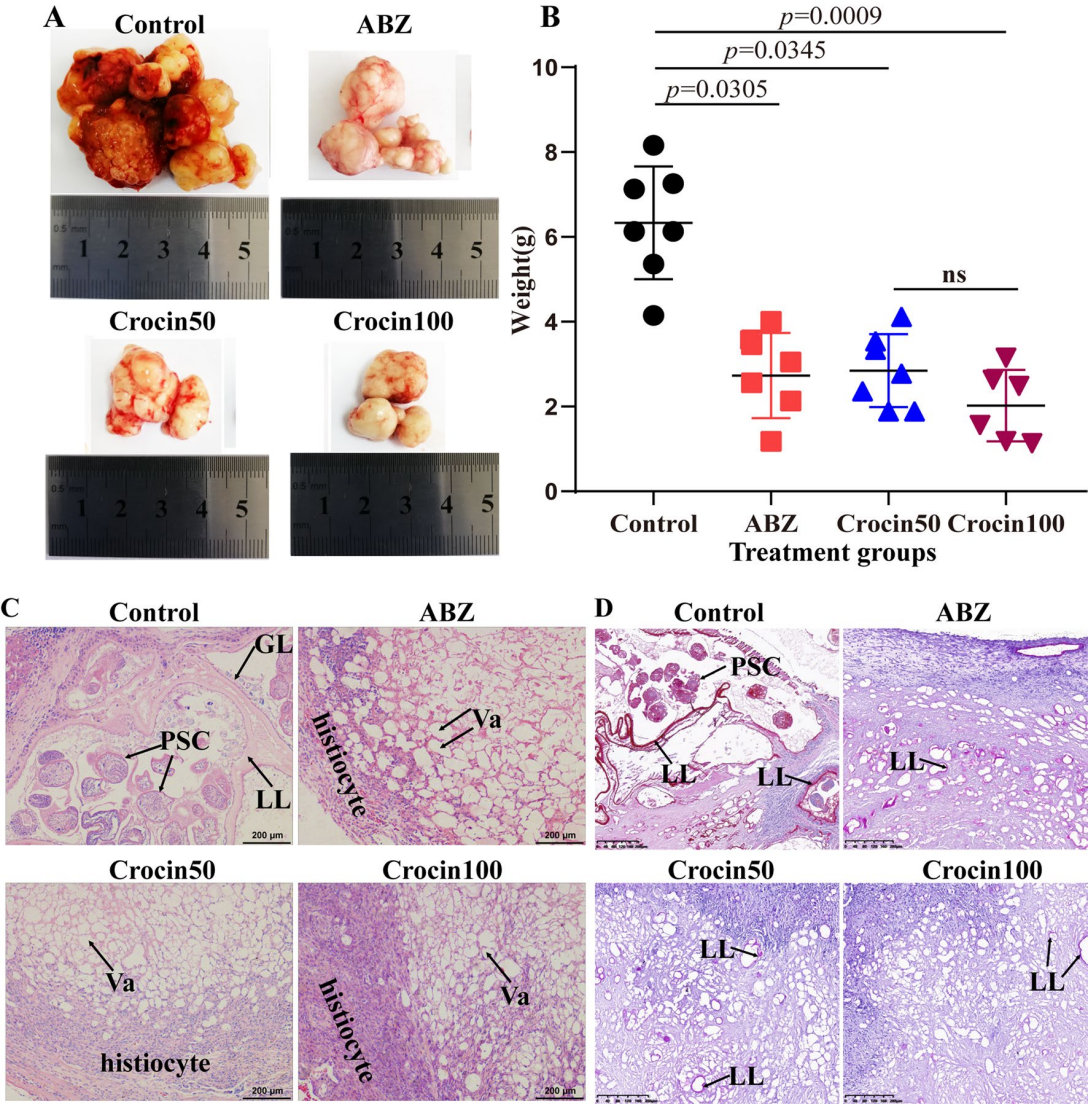
**a**–**d** In vivo activity of crocin against *E. multilocularis* metacestodes. Fourteen weeks after infection of BALB/c mice with protoscoleces, the mice were administered crocin intragastrically for 6 weeks. The positive control treatment was 100 mg/kg albendazole (*ABZ*). **a** Gross morphology of metacestodes. **b** Quantitative weight analysis of the metacestode masses. The data are presented as the mean ± SD. **c** *E. multilocularis* metacestodes were observed by HE staining. Protoscoleces were still observed in the control and ABZ groups.* Scale bar* = 200 μm.* FT* Fibrous tissue,* Va* vacuole. **d** LL PAS staining. The sections show PAS-positive LLs of *E. multilocularis* metacestodes.* Scale bar* = 200 μm. For other abbreviations, see Figs. 1 and 3

**Fig. 7:**
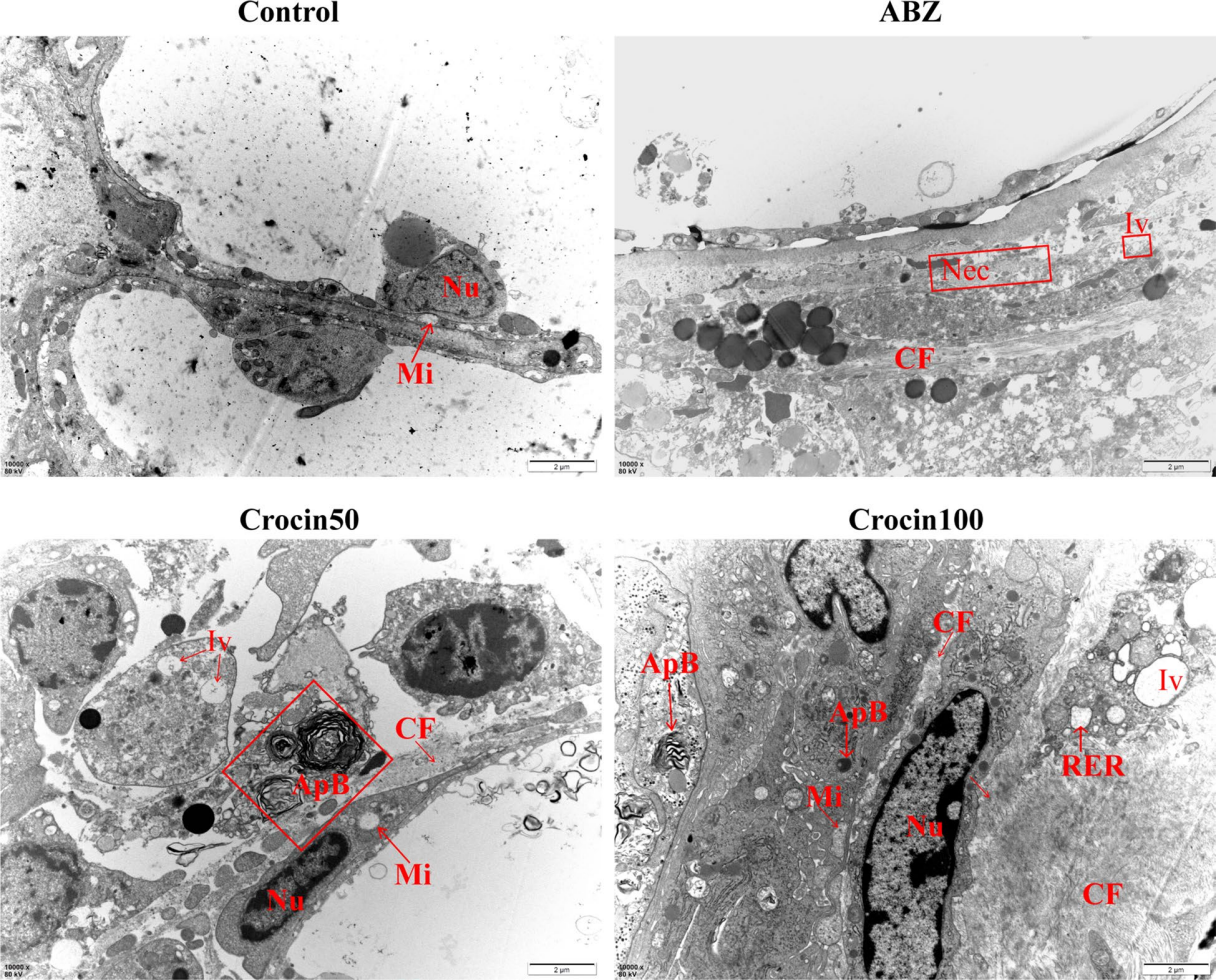
The host tissues around the metacestode were observed by TEM. *Nec* Necrosis,* Iv* intracytoplasmic vacuoles,* RER* rough endoplasmic reticulum,* CF* collagenous fibre.* Scale bars* = 2 μM. For other abbreviations, see Figs. 3, 5 and  6

The potential activity of crocin against *E. multilocularis* in vivo was assessed in mice infected with *E. multilocularis* protoscoleces. During the treatment, some animals died; the cause of death was confirmed by necropsy as asphyxia. No adverse reactions were observed in the other mice during the treatment. After 6 weeks, all mice were euthanized, and their metacestode tissues were carefully collected. Representative metacestode images are shown in Fig. 6a. The parasite weight data were not normally distributed according to the Shapiro–Wilk test (*W* = 0.91; *p* = 0.02). Kruskal–Wallis analysis indicated that the wet weights of the metacestodes were significantly lower in the Crocin50 group (2.85 ± 0.86 g), the Crocin100 group (2.02 ± 0.85 g) and the ABZ group (2.73 ± 1.01 g) than in the control group (6.33 ± 1.33 g) (Fig. 6b). The mass was surrounded by a layer of fibrous connective tissue that still contained portions of the laminated layer and germinal layer as well as some protoscoleces in the control group and ABZ group (Fig. 6c). In the crocin-treated group, only a few laminated layers were observed by PAS staining (Fig. 6d). After treatment with ABZ and crocin, significant collagen deposition was observed in host tissues around the metacestodes (Fig. 7). In addition, autophagosomes were also observed after crocin treatment (Fig. 7). Protoscoleces isolated from metacestodes in the control and ABZ groups were distinguished from the viable protoscoleces by eosin exclusion. However, crocin treatment reduced the number of protoscoleces isolated from the metacestodes, and the protoscoleces were stained with eosin (Additional file 1: Fig. S1). Furthermore, SEM was used to observe the microscopic changes in protoscoleces separated from metacestodes (Additional file 2: Fig. S2). The protoscoleces isolated from the control group and the ABZ group were invaginated, while those isolated from the crocin-treatment group exhibited contracted walls.

### Effect of crocin on cytokine expression

**Fig. 8:**
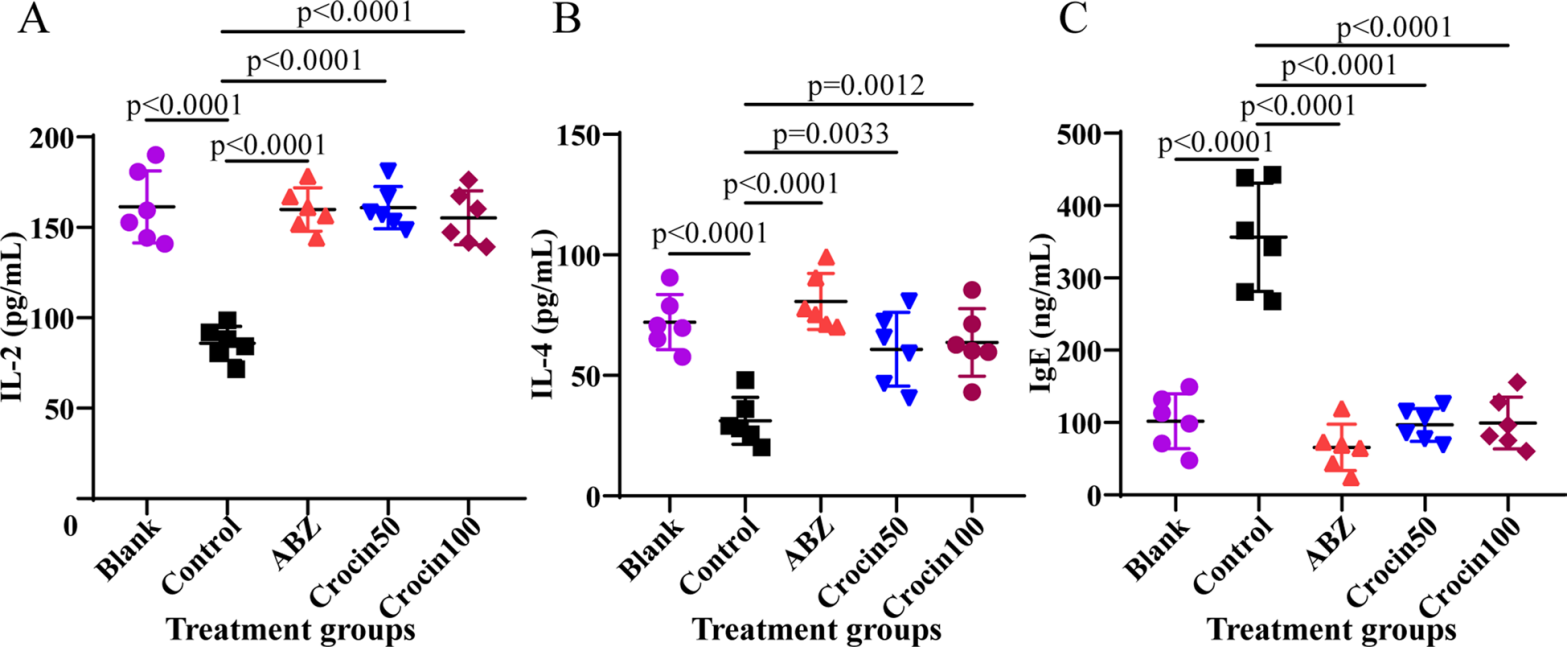
**a**–**c** Cytokine expression in serum was measured by enzyme-linked immunosorbent assay. Serum levels of interleukin (*IL*)-2 (**a**), IL-4 (**b**) and immunoglobulin E (*IgE*) (**c**) are shown. The data are presented as the mean ± SD. For other abbreviations, see Figs. 5 and  6

To analyse the immune effects induced by crocin, we used ELISA kits to examine the levels of the cytokines IL-2, IL-4, and IgE in serum (Fig. 8). Compared with the blank group, the control group exhibited lower IL-2 and IL-4 expression levels and higher IgE expression. However, crocin increased the expression of IL-2 and IL-4 in serum and decreased the expression of IgE in serum of the control group. In addition, ABZ increased the expression of IL-2 and IL-4 and decreased the expression of IgE in serum.

### Regulatory effects of crocin on the expression levels of MMP2 and MMP9

**Fig. 9:**
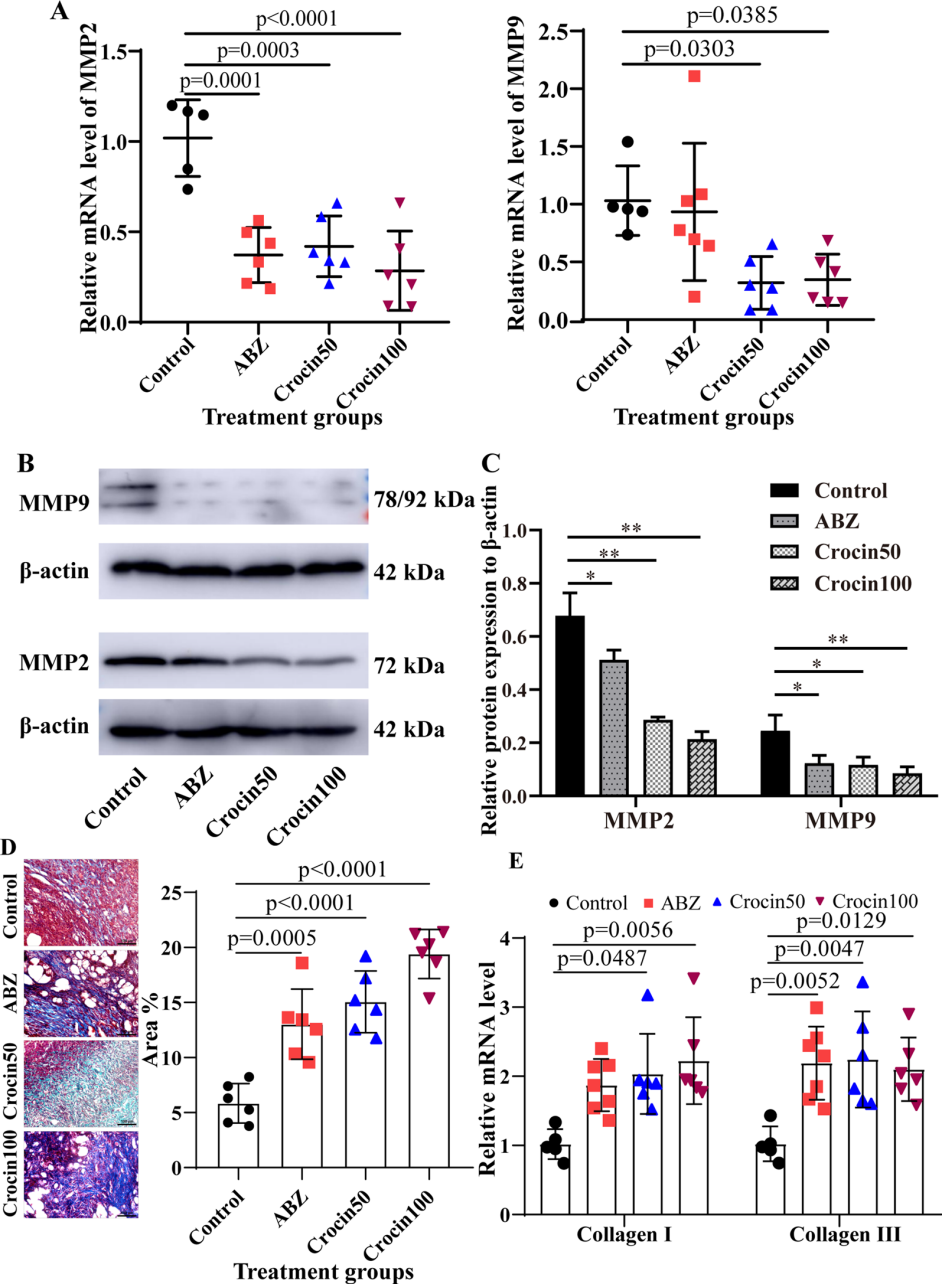
**a**–**e** Crocin inhibited the expression of matrix metalloproteinase (*MMP*) 2 and MMP9 in the host tissues around metacestodes. **a** Messenger RNA expression levels of MMP2 and MMP9 were analysed by real-time quantitative polymerase chain reaction (RT-qPCR). **b** Western blotting was used to detect the protein expression of MMP2 and MMP9. **c** Quantitative analysis of MMP2 and MMP9 protein levels. The data are presented as the mean ± SD of three experiments. **d** Metacestode sections were stained with Masson trichrome stain. The data are presented as the mean ± SD of six independent experiments.* Scale bar* = 100 μm. **e** The mRNA expression levels of collagen I and collagen III were analysed by RT-qPCR. The data are presented as the mean ± SD. For other abbreviations, see Figs. 5 and  6

Metacestodes were surrounded by host connective tissue that demonstrated a chronic granulomatous response. A large number of activated fibroblasts in the connective tissue layer can synthesize MMP2 and MMP9 and participate in the exogenous growth of metacestodes. Therefore, we examined whether crocin regulates the expression of MMP2 and MMP9. As shown in Fig. 9a, crocin downregulated the mRNA levels of MMP2 and MMP9 in the metacestode host tissues, but we did not identify a dose-dependent decrease. Similarly, the protein levels of MMP2 and MMP9 were downregulated (Fig. 9b, c).

The main function of MMPs is to decompose the extracellular matrix; therefore, we used Masson staining to investigate collagen synthesis in metacestodes. The results showed that collagen deposition increased in the metacestodes after crocin treatment (Fig. 9d). Type I collagen and type III collagen are major components of the extracellular matrix, and their production and renewal are related to fibrogenesis [28]. Therefore, we evaluated the effect of crocin on collagen mRNA levels. The results showed that the mRNA expression of type I and type III collagen was higher in crocin-treated metacestodes than in control metacestodes (Fig. 9e). These results demonstrated that crocin may affect the growth of metacestodes by inhibiting MMP2 and MMP9 expression in host tissue around the metacestodes.

## Discussion

AE is a zoonotic parasitic disease caused by the larvae of *E. multilocularis* that seriously affects the health of people in rural areas and causes considerable social problems and economic losses [29]. Current chemotherapies rely on benzimidazole treatment. However, benzimidazoles show parasitostatic effects rather than parasiticidal effects [30]. Thus, more effective drugs for AE need to be developed.

Recently, traditional Chinese medicines have been identified that are beneficial for the treatment of AE, including thymol and *Cnidium* extract [15, 31], which are considered nontoxic due to their chemotherapeutic activity. In the present study, we evaluated the in vitro efficacy of crocin against *E. multilocularis* metacestodes using PGI assays. The glycolytic enzyme PGI is a moonlighting protein that has been shown to stimulate the proliferation of both parasite germinal layer cells and mammalian cells [32]. PGI is also a prominent component of vesicle fluid and has been exploited for the development of PGI screening assays, which can be used to quantitatively screen for drugs that impair metacestode structural integrity in such a way that vesicle fluid is released into the medium supernatant [22]. Our results showed that crocin was significantly toxic to metacestodes and germinal cells, with an EC50 of 11.36 μM and an IC50 of 10.05 μM, respectively. Crocin was less toxic to mammalian cells than to *E. multilocularis* metacestodes and germinal cells. According to TEM observations, after crocin treatment, the metacestode vesicle germinal layer separated from the laminated layer, and part of the germinal layer structure was destroyed; only a few unconnected stem cells remained visible. This finding suggested that crocin potentially has therapeutic efficacy against *E. multilocularis* metacestodes.

The germinal layer of *E. multilocularis* metacestodes has a variety of cell types, including undifferentiated stem cells, glycogen storage cells, connective and muscle tissue cells, and nerve cells [33]. The germinal layer buds towards the inside, giving rise to brood capsules, which in turn generate protoscoleces. In the present study, high concentrations of crocin caused the death of all protoscoleces. The survival rate of the protoscoleces incubated with 10 μM crocin for 7 days was less than 40%. In addition, crocin was effective against protoscoleces in a dose-dependent manner. These results confirm that crocin has a parasiticidal effect. In addition, the anti-AE activity of the effective compounds in vivo was determined from parasite weight [8]. Crocin presented stronger protoscolicidal activity in vitro and obvious inhibitory effects against the growth of *E. multilocularis* metacestodes in experimentally infected mice.

During the onset of AE, there is an imbalance between activation of the T helper type 1 (Th1) cellular immune response and activation of the Th2 cellular immune response, leading to the differential release of cytokines. Here, we detected the serum levels of IL-2, IL-4 and IgE in mice. Compared with the control treatment, crocin administration enhanced the serum levels of IL-2 and IL-4 in mice in a dose-dependent manner. IL-4 is a vital immune factor that combats infection by extracellular parasites during the host immune response [34]. In the advanced stage of AE infection, a reduced level of IL-4 is conducive to the growth of AE metacestodes [35]. Therefore, the increase in the IL-4 level after crocin treatment was an important indicator of the therapeutic efficacy of crocin in AE-infected BALB/c mice at an advanced stage of infection. IL-2, an important mediator of inflammation and the immune response in a variety of infectious diseases, can enhance host immunity and inhibit the growth of tumours and parasites [36]. AE infection increases the expression of the IL-2 receptor and the consumption of IL-2, thereby inhibiting the immune activity of T lymphocytes and triggering the immune escape of parasites [37]. As a result, the increased level of IL-2 after crocin treatment indicated that crocin had enhanced the host’s immunity against *E. multilocularis*. During the growth of AE metacestodes, multiple antigens are produced that directly infiltrate the host tissues around the metacestodes [38]. Consequently, the relative levels of IgE, IgM and IgG are enhanced following AE infection [39]. Our data consistently showed that IgE levels were increased in the control group but reduced after crocin administration, suggesting the therapeutic efficacy of crocin against AE.

Parasite tissues produce continuously released metabolites that further interact with surrounding host tissues via the laminated layer [22]. Later on, collagen can accumulate around the lesions, giving rise to typical granulomas [40]. MMPs are a class of zinc-dependent proteases that are responsible for the degradation of the extracellular matrix in many tumours. Although AE growth is benign, its biological behaviour is malignant. Crocin significantly inhibits tumour growth [10], and it also downregulates MMP2 and MMP9 [10], which are involved in extracellular matrix degradation of tumour cells. In the current study, crocin administration downregulated the mRNA and protein levels of MMP2 and MMP9 in host tissues around metacestodes. In contrast, crocin treatment increased collagen deposition in host tissues around metacestodes. These changes can restrict the proliferation of *E. multilocularis* in the host mediated by endogenous and exogenous budding reproduction [41]. Infectious granulomas are formed by the interaction between *E. multilocularis* and the host. In addition to immune cell infiltration, abundant activated fibroblasts are distributed in the metacestode microenvironment [40]. These fibroblasts are the main sources of MMPs secreted in metacestodes.

Crocin is a water-soluble carotenoid that has the advantage of being able to be administered orally. A previous study also demonstrated a low IC_50_ of crocin (3 mg/mL) in hepatocytes [42]. In that study, crocin did not markedly damage any major organ in mice at acute toxicity (3 g/kg, administered intraperitoneally or orally) or subacute toxicity (less than 180 mg/kg) [43]. We also assessed the toxicity of crocin in mice and mammalian cells. Crocin did not influence routine blood indexes, liver function indexes or morphology. Crocin was verified to be safe for the treatment of AE. In fact, crocin presents obvious efficacy against liver and kidney fibrosis [44], and undoubtedly has a protective effect against AE infection-induced liver fibrosis [45].

## Conclusions

In conclusion, crocin is a promising alternative drug for the treatment of AE that protects against the activity of *E. multilocularis* both in vitro and in vivo with relatively low toxicity. Crocin downregulates MMP2/9 in host tissues surrounding metacestodes to inhibit the degradation of the extracellular matrix. Nevertheless, the immune effects of crocin in the treatment of AE need further study.

## Supplementary Information

**Additional file 1: Fig. S1. **Survival analysis of protoscoleces in metacestodes after crocin treatment. After 6 weeks of treatment, metacestodes were isolated from the abdominal cavity of mice. The metacestode of each mouse was cut into pieces by the same person, and the contents were filtered through gauze into a 50 ml centrifuge tube. The contents were centrifuged at 5000 rpm for 5 min, and the precipitation was stained with 0.1% eosin. The number of live and dead protoscoleces was counted under a microscope. Scale bar = 200 μm. **A** Protoscolex morphology isolated from mouse metacestodes. The blue arrows show the calcareous body, the red arrows show the live protoscoleces, and the black arrows show the dead protoscoleces. **B** Total number of protoscoleces. **C** The viability of the protoscoleces was assessed by eosin staining.

**Additional file 2: Fig. S2.** SEM observation of isolated protoscoleces from metacestode in mouse. In the control group and ABZ group, intact protoscoleces were observed, and the protoscoleces were invagination type. After treatment with crocin, the body wall of the protoscoleces appeared wrinkled. Representative images are displayed separately. Scale bar = 50 μm.

## Data Availability

Data supporting the conclusions of this article are included within the article.

## Abbreviations

ABZ: Albendazole
ABZSO: Albendazole sulphoxide
AE: Alveolar echinococcosis
cDNA: Complementary DNA
DMEM: Dulbecco’s modified Eagle medium
EC50: Median effective concentration
ELISA: Enzyme-linked immunosorbent assay
FBS: Foetal bovine serum
GAPDH: Glyceraldehyde-3-phosphate dehydrogenase
HE: Haematoxylin–eosin
HFF: Human foreskin fibroblast
IC50: Median inhibitory concentration
IgE: Immunoglobulin E
IL: Interleukin
MMP: Matrix metalloproteinase
mRNA: Messenger RNA
NS: Normal saline
PAS: Periodic acid Schiff
PBS: Phosphate-buffered saline
PGI: Phosphoglucose isomerase
RH: Reuber rat hepatoma
RT–PCR: Real-time polymerase chain reaction
SEM: Scanning electron microscopy
TEM: Transmission electron microscopy
Th: T helper cell
