# Supplementary material for: In vivo and in vitro efficacy of crocin against Echinococcus multilocularis
Source: Parasit Vectors. 2021 Jul 13;14:364. doi: 10.1186/s13071-021-04866-4 (PMC8278753; doi:10.1186/s13071-021-04866-4)
Supplement: Supplementary file 1 — Additional file 1: Fig. S1. Survival analysis of protoscoleces in metacestodes after crocin treatment. After 6 weeks of treatment, metacestodes were isolated from the abdominal cavity of mice. The metacestode of each mouse was cut into pieces by the same person, and the contents were filtered through gauze into a 50 ml centrifuge tube. The contents were centrifuged at 5000 rpm for 5 min, and the precipitation was stained with 0.1% eosin. The number of live and dead protoscoleces was counted under a microscope. Scale bar = 200 μm. A Protoscolex morphology isolated from mouse metacestodes. The blue arrows show the calcareous body, the red arrows show the live protoscoleces, and the black arrows show the dead protoscoleces. B Total number of protoscoleces. C The viability of the protoscoleces was assessed by eosin staining. [file 13071_2021_4866_MOESM1_ESM.doc]

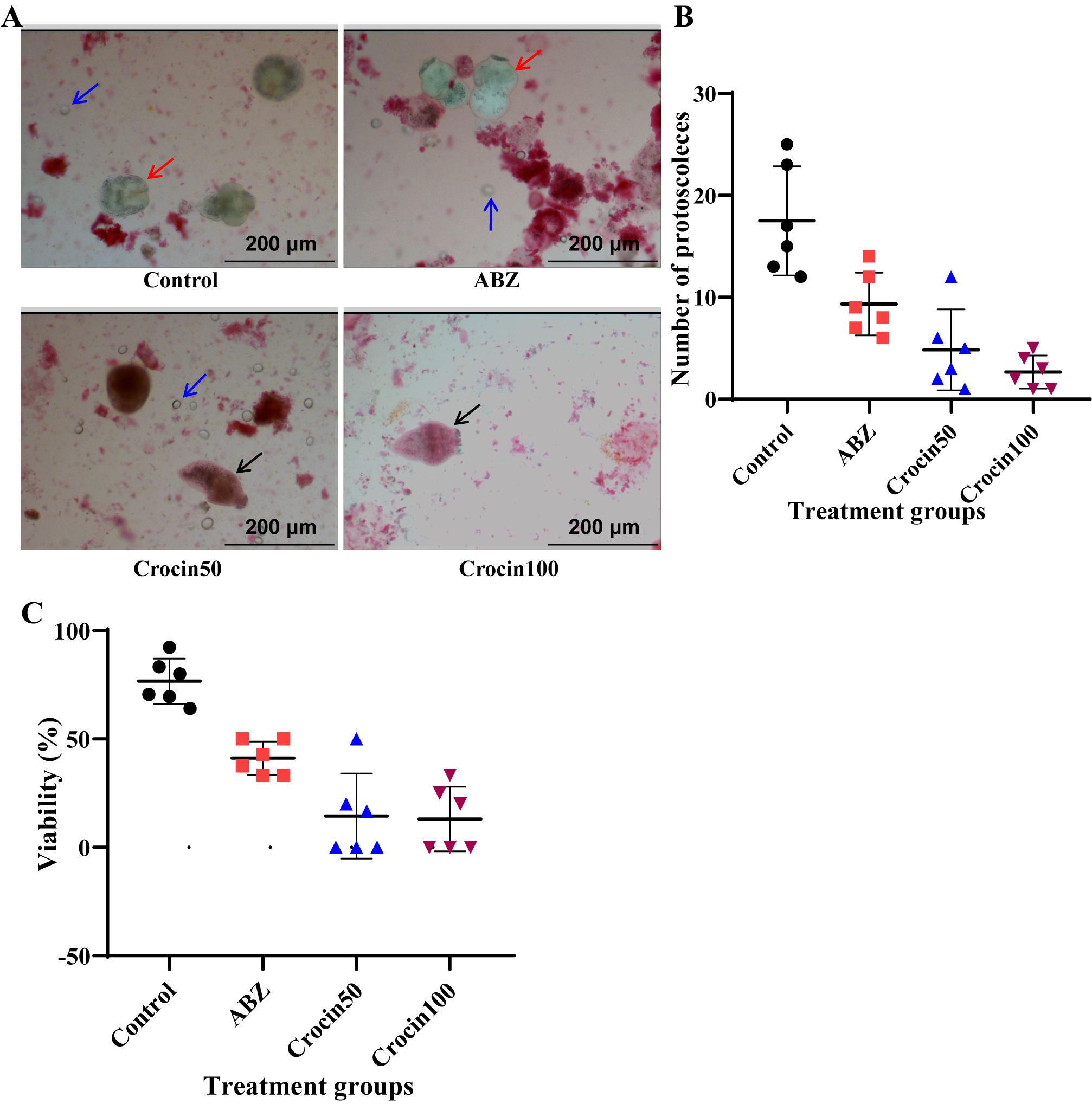


**Fig. S1 Survival analysis of protoscoleces in metacestodes after crocin treatment.** After 6 weeks of treatment, metacestodes were isolated from the abdominal cavity of mice. The metacestode of each mouse was cut into pieces by the same person, and the contents were filtered through gauze into a 50 ml centrifuge tube. The contents were centrifuged at 5,000 rpm for 5 min, and the precipitation was stained with 0.1% eosin. The number of live and dead protoscoleces was counted under a microscope. Scale bar = 200 μm. (**A**) Protoscolex morphology isolated from mouse metacestodes. The blue arrows show the calcareous body, the red arrows show the live protoscoleces, and the black arrows show the dead protoscoleces. (**B**) Total number of protoscoleces. (**C**) The viability of the protoscoleces was assessed by eosin staining.
