# Supplementary material for: In vivo and in vitro efficacy of crocin against Echinococcus multilocularis
Source: Parasit Vectors. 2021 Jul 13;14:364. doi: 10.1186/s13071-021-04866-4 (PMC8278753; doi:10.1186/s13071-021-04866-4)
Supplement: Supplementary file 2 — Additional file 2: Fig. S2. SEM observation of isolated protoscoleces from metacestode in mouse. In the control group and ABZ group, intact protoscoleces were observed, and the protoscoleces were invagination type. After treatment with crocin, the body wall of the protoscoleces appeared wrinkled. Representative images are displayed separately. Scale bar = 50 μm. [file 13071_2021_4866_MOESM2_ESM.doc]

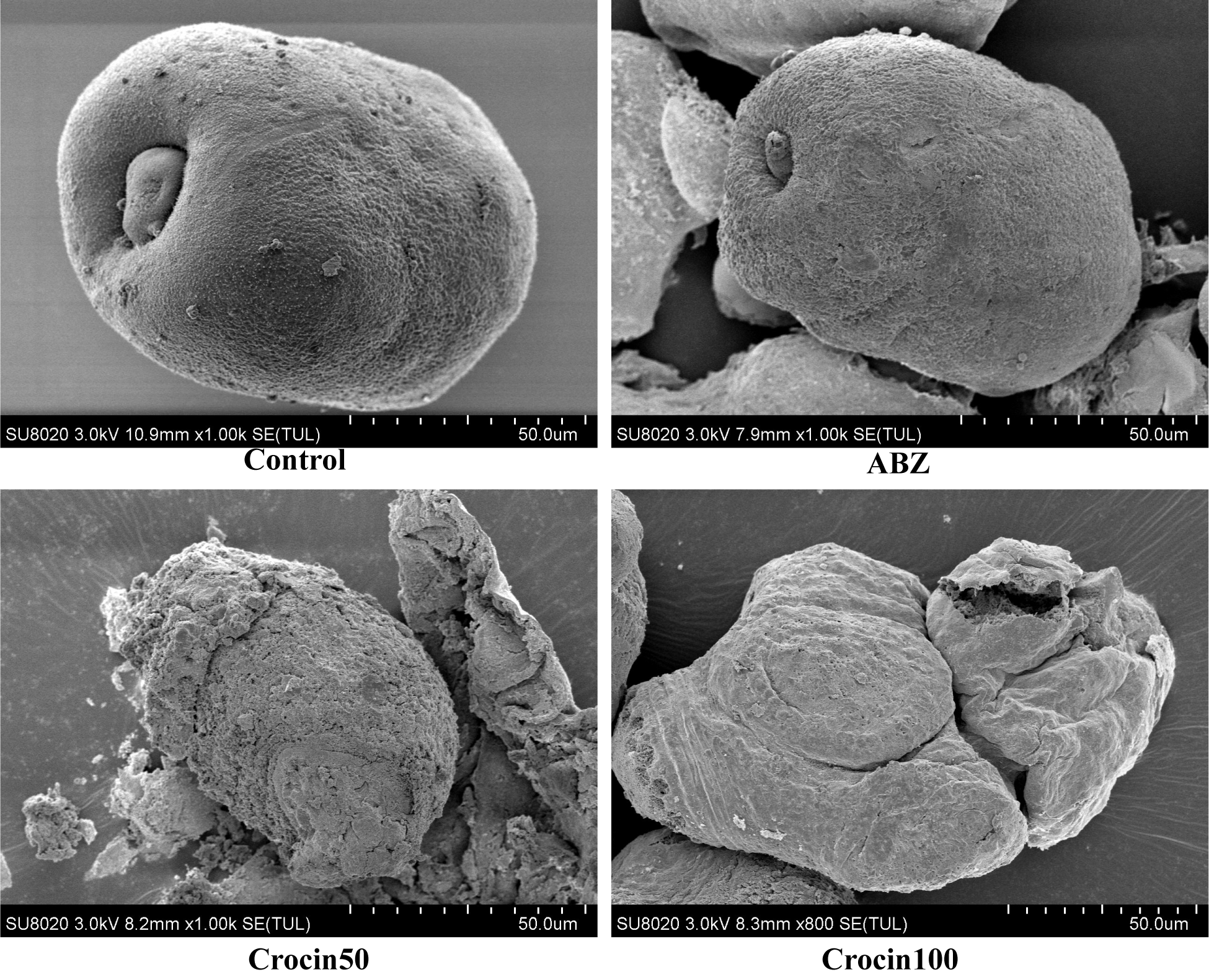
**Fig. S2 SEM observation of isolated protoscoleces from metacestode in mouse.** In the control group and ABZ group, intact protoscoleces were observed, and the protoscoleces were invagination type. After treatment with crocin, the body wall of the protoscoleces appeared wrinkled. Representative images are displayed separately. Scale bar = 50 μm.
